# Ubiquitous protein lactylation in health and diseases

**DOI:** 10.1186/s11658-024-00541-5

**Published:** 2024-02-05

**Authors:** Junyong Wang, Ziyi Wang, Qixu Wang, Xiao Li, Yaping Guo

**Affiliations:** 1 Department of Pathophysiology, School of Basic Medical Sciences, Zhengzhou University, Science Avenue 100, Zhengzhou, 450001 Henan China; 2 State Key Laboratory of Esophageal Cancer Prevention and Treatment, Zhengzhou, 450001 Henan China; 3 Department of Gastroenterology, Henan Provincial People’s Hospital, Zhengzhou University People’s Hospital, Zhengzhou, 450001 Henan China; 4 Center for Basic Medical Research, Academy of Medical Sciences, Zhengzhou University, Zhengzhou, 450001 Henan China

**Keywords:** Glycolysis, Lactate, Lactylation, Pathological process

## Abstract

For decades, lactate has been considered a byproduct of glycolysis. The lactate shuttle hypothesis shifted the lactate paradigm, demonstrating that lactate not only plays important roles in cellular metabolism but also cellular communications, which can transcend compartment barriers and can occur within and among different cells, tissues and organs. Recently, the discovery that lactate can induce a novel post-translational modification, named lysine lactylation (Kla), brings forth a new avenue to study nonmetabolic functions for lactate, which has inspired a ‘gold rush’ of academic and commercial interest. Zhang et al. first showed that Kla is manifested in histones as epigenetic marks, and then mounting evidences demonstrated that Kla also occurs in diverse non-histone proteins. The widespread Kla faithfully orchestrates numerous biological processes, such as transcription, metabolism and inflammatory responses. Notably, dysregulation of Kla touches a myriad of pathological processes. In this review, we comprehensively reviewed and curated the existing literature to retrieve the new identified Kla sites on both histones and non-histone proteins and summarized recent major advances toward its regulatory mechanism. We also thoroughly investigated the function and underlying signaling pathway of Kla and comprehensively summarize how Kla regulates various biological processes in normal physiological states. In addition, we also further highlight the effects of Kla in the development of human diseases including inflammation response, tumorigenesis, cardiovascular and nervous system diseases and other complex diseases, which might potentially contribute to deeply understanding and interpreting the mechanism of its pathogenicity.

**Graphical Abstract:**

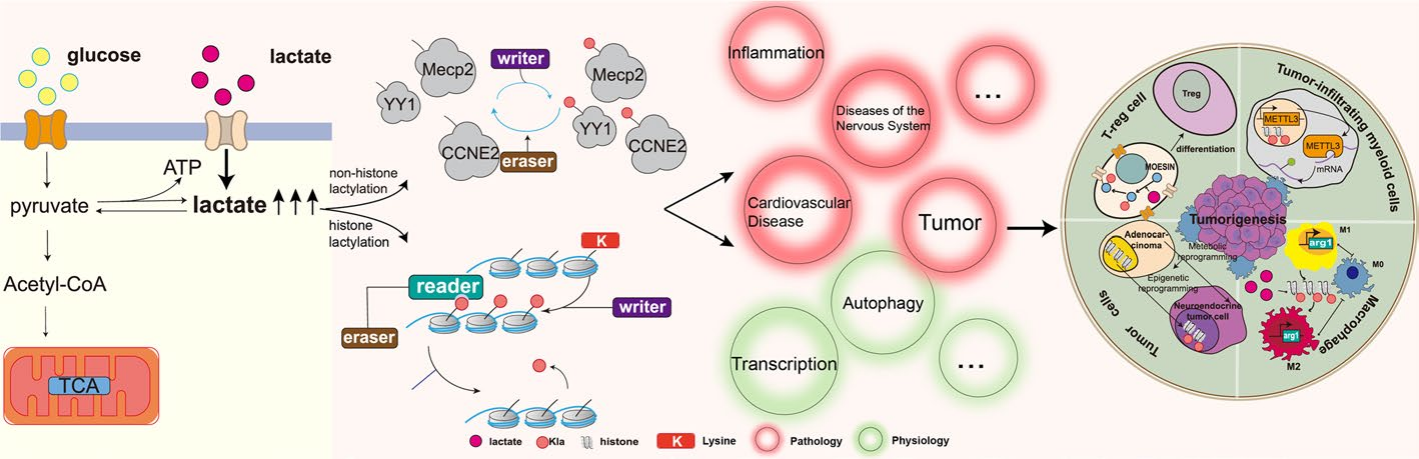

## Introduction

Metabolism is fundamental for living systems. To promote growth and survival, cells take up and metabolize diverse nutrients in the surrounding environment to sustain cellular energy demands. Glucose, as a major energy source, is metabolized into pyruvates, which are subsequently converted into acetyl-CoA in the mitochondria for efficient energy production in aerobic condition or lactate in the cytoplasm under hypoxia [1]. Although the functions of the former have been well documented, lactate has long been considered as a metabolic waste product when cells are faced with hypoxia [2]. However, mounting evidences have shown otherwise. Based on the lactate shuttle hypothesis, lactate acts as the principal messenger in delivery of oxidative and gluconeogenic substrates as well as in cell signaling [3]. Brooks proposed that lactate can be produced under fully aerobic conditions and utilized in diverse cells to fulfill several purposes including a major energy source, the major gluconeogenic precursor and a signaling molecule [4]. For example, lactate is actually a source of energy and can meet the brain’s excitatory activity [5] when blood glucose levels are insufficient. It is the primary fuel for the tricarboxylic acid (TCA) cycle [6]. At the same time, lactate serves as a redox signaling molecule between cells and tissues, playing an important role in cellular metabolism [7].

The repeated utilization of lactate by different cell populations in the tumor microenvironment (TME) is known as metabolic symbiosis [8]. In 1920s, Otto Warburg found that glucose can indeed be incompletely oxidized to generate the metabolite lactate instead of complete oxidization product CO_2_, even in the presence of oxygen, through a phenomenon known as Warburg effect or aerobic glycolysis [9]. The Warburg effect describes the enhanced lactate production in cancer, where cancer cells tend to produce lactate through glycolysis rather than oxidative phosphorylation even in the presence of sufficient oxygen, which may be related to the abnormal proliferation of cancer cells [10]. Additionally, a number of researchers proposed the reverse Warburg effect, a novel model of “two-compartment metabolic coupling”. In this process, cells in the TME produce high levels of energy-rich fuels such as pyruvate, fatty acids, and lactate, which in turn can be used for mitochondrial OXPHOS in tumor cells and for efficient ATP production. Meanwhile lactate metabolism coupling implies that high levels of lactate not only have an effect on tumor cells, but also on other cells within the TME [11, 12]. Besides, overwhelming evidences demonstrate that the phenomenon of Warburg effect which leads to lactate production can be observed in a variety of noncancerous diseases such as pancreatitis [13, 14], sepsis [15], atherosclerosis [16–18], myocardial infarction [19, 20] and heart failure [21]. Furthermore, cells can continuously carry out aerobic glycolysis to produce lactate when cells encounter various stressful conditions, such as chronic exposure to high altitude, trauma and so on [22]. In summary, in contrast to the early opinion as simply a metabolic byproduct, lactate is the inexorable end product of glycolysis and exerts multiple regulatory functions in diverse cells.

In 2019, Zhang et al. discovered a novel function for lactate, termed lysine lactylation (Kla), which represents a novel metabolite-derived post-translational modification (PTM) like acetylation, and is manifested in histones as a new epigenetic mark [23]. Biochemically, lactylation occurs on the lysine residues of proteins by addition of a lactyl group with a mass shift of 72.021 Da, which was detected by liquid chromatography-tandem mass spectrometric (MS/MS) [23]. In 2022, Wan et al. discovered a signature fragment ion, the cyclic immonium occurred in MS/MS spectra of Kla-containing peptides, which can be used for robust Kla detection [24]. Similar to other epigenetic mark, the role of histone Kla was first described that can directly stimulate gene expression from chromatin [23]. Currently, histone Kla has been observed in many types of cells, predominantly in tumor cells and innate immune cells. In addition to histones, mounting evidences suggest that the non-histone proteins can be modified by Kla. For example, Yang et al. reported that macrophages can take up extracellular lactate to promote high mobility group box-1 (HMGB1) Kla during polymicrobial sepsis, which can further induce the endothelial barrier dysfunction [15]. Besides, autophagy, a conserved lysosomal degradation process has been found to be regulated by Kla [25]. Mechanistically, UNC-51-like kinase 1, a core autophagy protein, can phosphorylate the key glycolytic enzyme lactate dehydrogenase A (LDHA) and increase its enzyme activity, then promoting the production of lactate, which drives the Kla of vacuolar protein sorting 34 (Vps34). Vps34 Kla can not only facilitate autophagosome formation and maturation but also enhance endosome-lysosomal degradation that might participate in skeletal muscle homeostasis and cancer progression [26]. Taken together, Kla is widespread both in histone and non-histone proteins, and faithfully regulates various biological processes, such as transcription, metabolism and inflammatory responses.

**Fig. 1:**
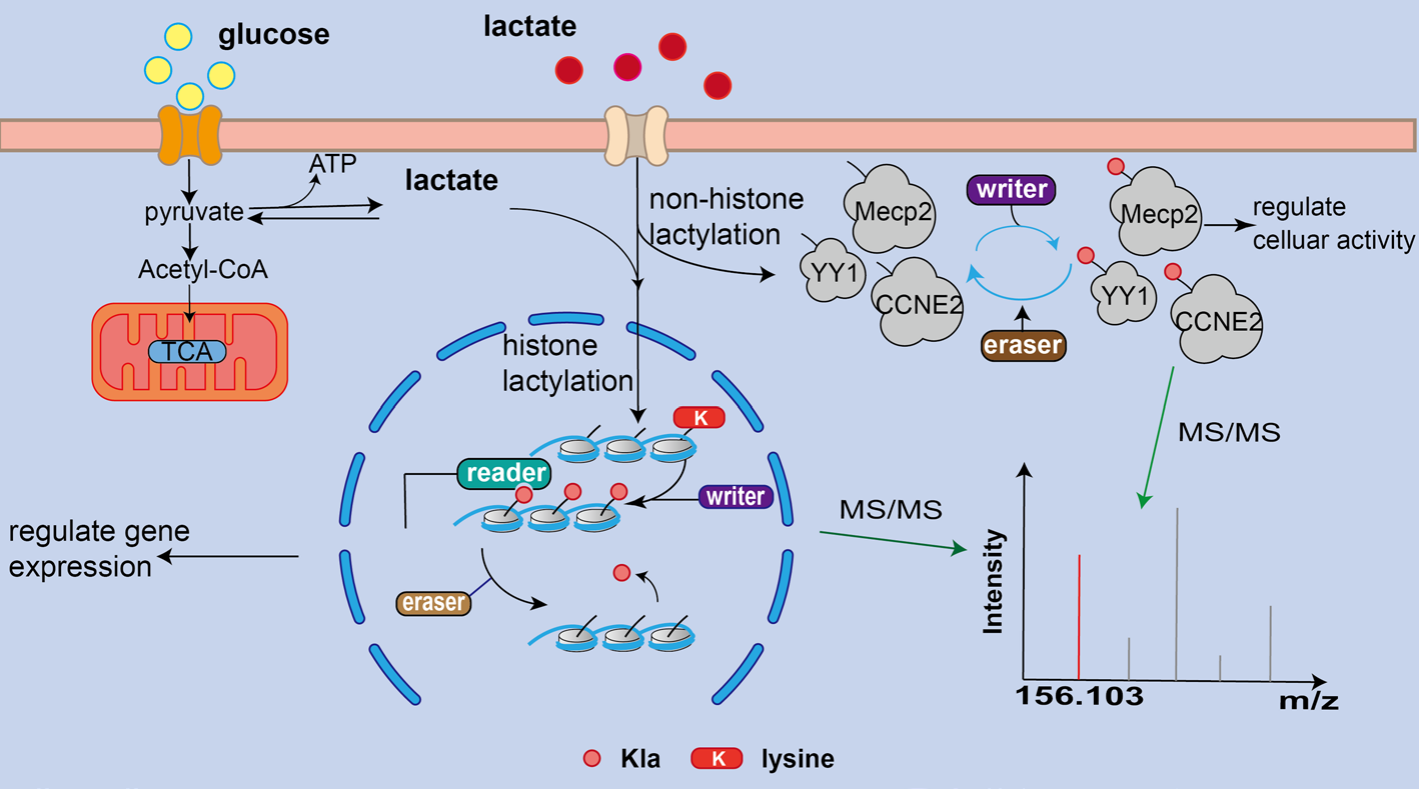
The process of lactate production and Kla in cells. Glycolysis produces lactate and extracellular lactate molecules can enter the cell. The lactate is involved in Kla of histones or non-histone proteins with the help of related enzymes. What’s more, the datasets of lactylome are determined by MS/MS based proteomics technology

Moreover, the advances in MS/MS-based proteomics technology have enabled to detect an amount of lactylated proteins and sites in many species [27], including eukaryotes such as *Homo sapiens, Rattus norvegicus, Oryza sativa, Botryits cinerea* and prokaryotes like *Escherichia coli (E. coli)*, which are also referred as lactylome. All in all, since Kla is firstly discovered in 2019, an amount of lactylated proteins and sites has been identified, which provides a launch point for a deeper investigation into the important roles of Kla. Meanwhile, mounting researches were carried out to experimentally investigate the regulatory mechanism and functional consequence of Kla. Similar to other reversible PTMs, Kla is catalyzed by two types of enzymes which specifically add or remove Kla, called the writer and eraser for Kla, respectively [28]. For example, Dong et al. identified YiaC as a lactylase and CobB as a delactylase in *E. coli* [29] (Fig. 1). Thus, Kla of proteins is precisely regulated in vivo, whereas dysregulation of this processes is intricately linked to many complex diseases such cardiovascular diseases and cancer. Here, in this review, we comprehensively reviewed and curated the existing literature to retrieve the newly identified Kla sites on both histones and non-histone proteins and summarized recent major advances toward its regulatory mechanism. We also thoroughly investigated the function and underlying signaling pathway of Kla and further highlighted the effects of Kla in the development of human diseases.

### Ubiquitous protein lactylation

Zhang and his colleagues conducted a pioneering research and firstly proposed Kla as a novel histone mark in 2019. In the meanwhile, 28 Kla sites were detected on the core histones in cells from human and mouse. They also found that histone Kla level is usually in parallel with the lactate production and illustrated that both exogenous and endogenous lactate can directly contribute to Kla of histones. In other conditions such as hypoxia, adding the inhibitor of the mitochondrial respiratory chain complex I rotenone, glucose supplementation and M1 macrophage polarization, where cells tend towards enhanced glycolysis to some extent, resulting in producing lactate in cells, Kla can be also promoted. Conversely, they also demonstrated that histone Kla induced by hypoxia can be attenuated by the LDH inhibitor Oxamate and the PDK1 inhibitor DCA, whereas is fully suppressed when deleting both lactate dehydrogenase LDHA and LDHB, which both can inhibit lactate production. Together, this pioneering research demonstrate that lactate-derived histone Kla as a new epigenetic modification [23].

**Fig. 2:**
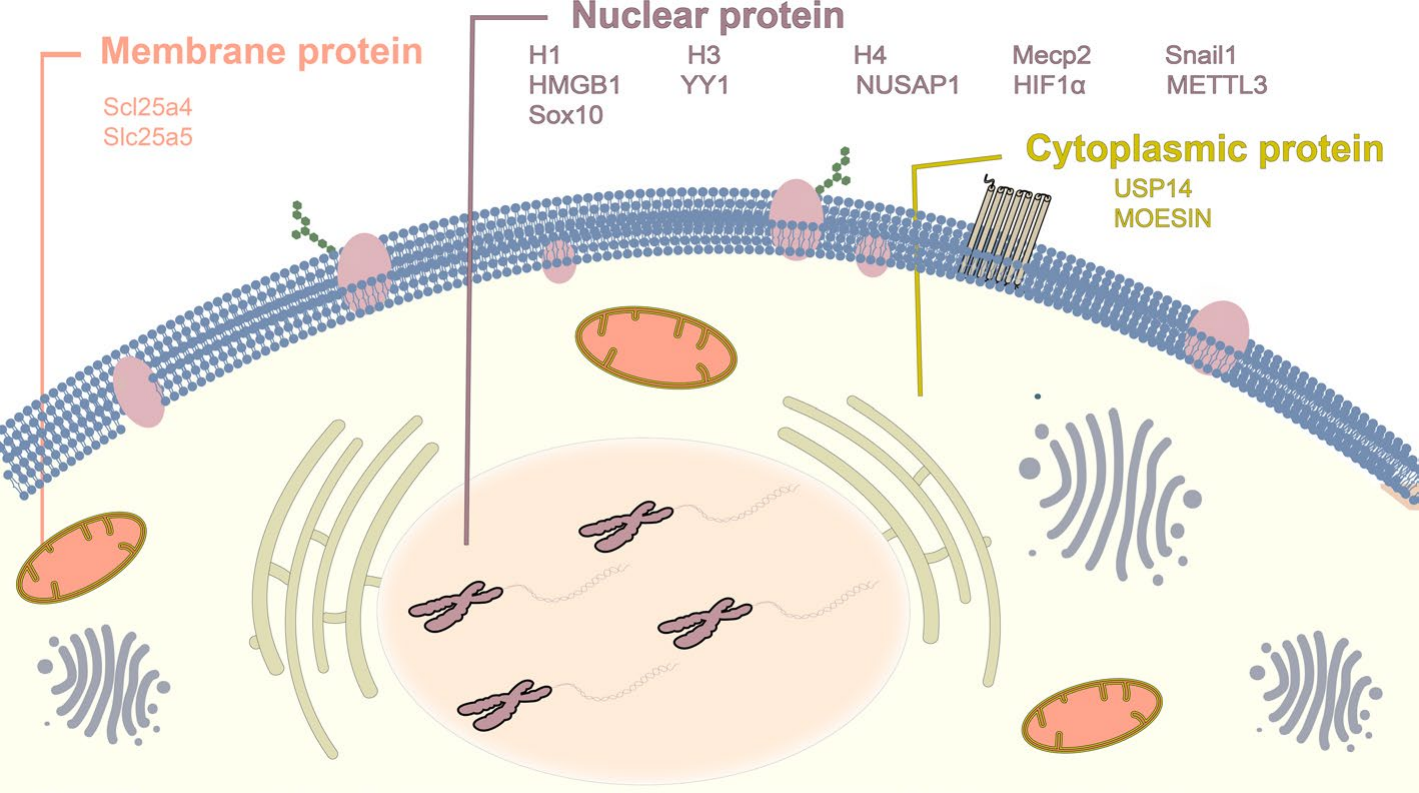
Protein Kla is widespread in cells. In addition to histones, certain non-histone proteins in the nucleus can also be lactylated. Kla is also present on some membrane proteins, and in the cytoplasm, there are proteins involved in Kla. The figure shows a list of proteins that can be lactylated in different subcellular localization of the cell

In addition to histones, certain nonhistone proteins can also be lactylated, such as K102 and K116 of human nucleolin [24]. And the ubiquitous nuclear protein, HMGB1 can also be modified by Kla, which further might be released from macrophages via exosome secretion [15]. Additionally, transcriptional regulators such as methyl-CpG-binding domain protein 2 and general transcription factor II-I was found to be lactylated, which might provide important extra evidence that Kla links cellular metabolism to gene regulation [24]. The transcription factor, Yin Yang-1 (YY1) was also found to bear increased Kla in the context of increased lactate under hypoxia, which can further enhance FGF2 transcription and promote angiogenesis [30]. Moreover, a key modulator of the transcriptional response to hypoxic stress, HIF1α can also undergo Kla when lactate imported into prostate cancer (PCa) cells via MCT1, which can stabilize HIF1α under normal hypoxic conditions, enhance KIAA1199 transcription and promote angiogenesis in PCa [31]. Hypoxia-induced glycolysis also promotes Kla of β-catenin, which promotes cell proliferation in colorectal cancer (CRC) by enhancing protein stability and expression of β-catenin [32]. Gu et al. demonstrated that lactate can facilitate the lactylation of MOESIN at K72 to regulate the development and immunosuppressive function of Regulatory T (Treg) cells [33]. Of note, enzymes which are involved in metabolic process, especially glycolysis, can be commonly modified by Kla [24]. For example, fructose bisphosphate aldolase A (ALDOA) is lactylated at K147 residue, which is conserved in human and mouse. The lactylation at K147 in turn can reduce ALDOA enzymatic activity, perhaps forming a negative feedback loop by which cell can decrease the excessive lactate levels [24]. Besides, another glycolytic enzyme, α-enolase was also identified to undergo lactylation at K343, which might impact the interaction of substrate–enzyme. Notably, it is found that dehydrogenase reductase 7, an enzyme that can catalyze various substrates, including steroid hormones, lipids and other metabolites, can be lactylated at K321 and this phenomenon is prevalent in different human tissues, such as the retina, spinal cord, liver, testis, ovary and prostate [24] (Fig. 2).

Furthermore, proteomics studies carried out in many species or cell lines have generated large volumes of Kla datasets over the years. Here, we summarized the Kla sites that have been identified in recent years including human, mouse, protozoa, plant and microbe. Firstly, in a comprehensive analysis of gastric cancer (GC) cells, 2375 Kla sites have been identified, indicating significant enrichment of these proteins in spliceosome function [34]. In order to investigate the role of Kla in hepatocellular carcinoma (HCC), Wu firstly collected three normal liver samples, three HCC samples (with no metastasis during 3 years of follow-up) and three lung metastasis samples of HCC, and then conducted the lactylome analysis. Totally, 2045 Kla sites on 960 proteins were identified. They found many of these lactylated proteins exhibits differential Kla level among the three different groups and were involved in diverse biological processes, such as amino acid metabolism, fatty acid metabolism and ribosomal protein synthesis [35]. Yang et al. demonstrated a global lactylome profiling on a collected HCC cohort, and 9275 Kla sites were identified by analyzing the proteome of the tumor and adjacent liver. Of these, 9256 sites are on non-histone proteins, showing that Kla is a universal modification beyond histone and transcriptional regulation [36]. Besides, the whole lactylome was also performed on tendon samples collected from patients with rotator cuff tendinopathy. Among 284 proteins, 872 Kla sites were identified. Compared to healthy individuals, 136 Kla sites of 77 proteins were found to be upregulated in pathological tendon, while 56 sites of 32 proteins were downregulated [37]. These lactylated proteins are tightly associated with cholesterol metabolism and synthesis of the fascial matrix. In 2023, Wang et al. investigated the lactylome of retinal microglia under hypoxia and identified a total of 3093 Kla sites on 751 proteins, including 77 sites of 67 proteins with increased Kla under the hypoxic condition. They also found that hyper Kla of microglia can promote angiogenesis and play important roles in retinal neovascularization [38]. In 2023, a study profiled Kla sites of human lungs under normal physiological conditions and identified 724 Kla sites on 451 proteins [39]. The Kla sites of these proteins are distributed throughout the body and have varying degrees of involvement in various biological processes.

With the advances in MS/MS-based proteomics technology, the lactylome of other species were successively identified. By evaluation of Kla of cortical proteins in a *Rattus norvegicus* model of cerebral ischemia–reperfusion injury (CIRI), a total of 1003 Kla sites were identified on 469 proteins [40]. In 2021, Zhang et al. conducted the first exhaustive proteome-wide survey of lactylated sites in *Trypanosoma brucei* and identified 387 Kla sites in 257 proteins [41]. In 2022, Zhao et al. identified 983 Kla sites on 523 lactylated proteins in total proteins extracted from *Toxoplasma gondii* (*T. gondii*) [42]. What’s more, Meng et al. reported the first global lactylome profiling in rice, identifying 638 Kla sites on 342 proteins in *Oryza sativa* [43]. In 2023, Shi et al. studied and analyzed lactate and protein Kla in *Zea mays* roots, identifying 37 Kla sites on 16 histones in the *Zea mays* [44]. Meanwhile, Wu et al. showed the first global acetylome, 2-hydroxyisobutyrylome and lactylome in sugarcane and a total of 215 Kla sites were identified in 139 modified proteins [45]. Additionally, Gao et al. conducted the first proteomic survey of Kla sites in the *Botryotinia fuckeliana*, identifying 273 Kla sites in 166 proteins [46]. In 2022, An et al. also discovered 1,458 Kla sites in 469 proteins from *Frankliniella occidentalis* (*F. occidentalis*) by comprehensive analysis of the lactylome in *F. occidentalis* [47]. Meanwhile, Yin et al. identified 1,964 Kla sites on 955 proteins of the *T. gondii* RH strain [48]. Song et al. performed a global lactylome analysis of *Phialophora verrucosa* and totally found 636 Kla sites of 420 proteins [49]. Moreover, Wang et al. mapped the proteome and lactylome of the *Nannochloropsis oceanica* and identified 868 Kla sites in 379 proteins [50]. Here, we also summarized well-known lactylome datasets under physiological and pathological conditions in diverse species, which were identified using the MS/MS-based proteomics technology (Table 1).

**Table 1:** We summarized the identified Kla sites and the corresponding proteins in different conditions and species

Conditions	Proteins	Sites	Tissue/cell	Species	References
Pathology	1014	2375	GC AGS cells	*Homo sapiens*	[34]
	960	2045	HCC	*Homo sapiens*	[35]
	N/A	9275	HCC	*Homo sapiens*	[36]
	284	872	Tendon	*Homo sapiens*	[37]
	751	3093	Microglia	*Homo sapiens*	[38]
	469	1003	Brain	*Rattus norvegicus*	[40]
Physiology	451	724	Lung	*Homo sapiens*	[39]
	257	387	N/A	*Trypanosoma brucei*	[41]
	523	983	N/A	*Toxoplasma gondii*	[42]
	342	638	Rice grains	*Oryza sativa*	[43]
	16	37	Maize roots	*Zea mays*	[44]
	139	215	N/A	*Saccharum hybrid*	[45]
	166	273	N/A	*Botryotinia fuckeliana*	[46]
	469	1458	N/A	*Frankliniella occidentalis*	[47]
	955	1964	N/A	*Toxoplasma gondii*	[48]
	420	636	N/A	*Phialophora verrucosa*	[49]
	379	868	N/A	*Nannochloropsis oceanica*	[50]

Due to data accumulation, computational prediction of Kla from protein sequences is an alternative approach to efficiently prioritize highly potential candidates for further experimental consideration. In this regard, there were 2 tools developed for predicting Kla, including FSL-KLA [51] and Auto-Kla [52]. In 2021, Jiang et al. constructed the first Kla benchmark dataset and developed a few-shot learning-based architecture method to predict the Kla sites on proteins. Based on this benchmark dataset, they designed a prediction model which can be used to generate useful candidates for further experiments [51]. In 2023, Lai and Gao proposed an efficient and accurate prediction model based on automated machine learning, called Auto-Kla, which can quickly and accurately discriminate Kla sites [52]. Indeed, identification of Kla sites through experiments is labor-intensive and time-consuming, whereas the aforementioned researches greatly facilitate the study of Kla by providing convenient and cost-effective predictors.

Although many studies demonstrated that the level of Kla in vivo is sensitive to cellular lactate and is quite different under diverse conditions, enzymes directly regulating reversible Kla, often called writers (catalyzing Kla), readers (recognizing Kla), and erasers (removing Kla), have not been fully identified and confirmed. Zhang et al. found that HAT p300 was determined potentially a histone Kla writer, which can catalyze and add the lactyl group from lactyl-CoA to the specific lysine sites [23]. Later, p300 was verified to act as the acyltransferase for lactylation on K1897 of α-MHC [21]. In contrast, Moreno-Yruela et al. also conducted a pioneering work to uncover the histone delactylases enzymes and found that HDAC1–3 and SIRT1–3 can act as delactylases to cleave the Kla marks [53]. Subsequently, Studied also demonstrated that SIRT1 can delactylase α-MHC [21], whereas SIRT3 can mediate the delactylation of cyclin E2 (CCNE2) and regulate Kla levels of CCNE2 [54]. Moreover, Dong et al. found that YiaC can act as the writer to catalyze the addition of Kla, whereas CobB can act as eraser to remove this modification in *E. coli* [29]. Together, the discovery of widespread Kla sites and their regulatory enzymes greatly promoted the process to explore functional Kla events which are precisely orchestrated to regulate various biological processes.

### The roles of lactylation in normal physiological states

Besides aerobic glycolysis in cancer, mounting evidences showed that even in resting humans or during exercise, lactate production occurs continuously, which might indicate that the accumulated lactate in normal physiological states can also act as a donor to promote Kla to participate in diverse cellular processes. In 2021, Yang et al. studied the distribution of Kla at K23 and K18 on histone H3 (H3K23la and H3K18la), and pan-histone Kla in mouse oocytes and pre-implantation embryos, respectively. They found that all three types of modification were enriched in GV-stage oocytes, and that H3K23la and pan-histone Kla could be detected in MII-stage oocytes, whereas H3K18la could not be detected. Additionally, the level of H3K23la, H3K18la and pan-histone was found to be decreased in zygotes after fertilization. It was also experimentally noted that hypoxic in vitro culture decreases histone Kla and thus hampers embryonic development in pre-implantation embryos in mice [55]. In 2022, Yang et al. demonstrated lactate can induce endometrial H3K18la, which further participates in endometrial remolding to facilitate the successful implantation. Finally, experiments also suggested that lactate-induced H3K18la in the endometrium seems to be common in all species [56]. In embryonic stem cells, lactate can also enhance H3K18la, which in turn facilitates transcriptional elongation of downstream target genes [57]. In addition, Lin et al. found a relationship between Kla and meiosis in mouse oocytes, suggesting that Tfap2a overexpression dramatically increased P300 expression, which in turn increased the levels of histone Kla including Pan Kla, H3K18la, and K12 Kla of histone H4 (H4K12la), ultimately impeding mouse oocyte meiotic progression [58]. This might suggest that H3K18la can serve as a histone mark for promoter activation and enhancement of tissue-specific activity and play an important role in embryonic formation and development [59]. Research has indicated that H3K18la can also serve as a potential biomarker for the diagnosis and prediction of the severity of septic shock [60]. Moreover, in bone remodeling, Kla levels are associated with osteoblast differentiation and bone formation. LDHA expression and intracellular lactate levels are increased during osteoblast differentiation, which is also accompanied by an increased the level of H3K18la. Mechanistically, depletion of LDHA significantly reduce H3K18la levels and further impaired osteogenic differentiation. In the meanwhile, JunB expression is also regulated by LDHA via H3K18la [61].

In addition, Kla was found to be closely related to exercise and some other aspects. During exercise, a large amount of lactate is produced, which in turn can induce Kla. After high intensity interval training, the Kla levels of protein in skeletal muscle and liver reached a peak at 24 h and they are potentially involved in the TCA, glycolysis, gluconeogenesis, pyruvate metabolism and oxidative phosphorylation pathways [62, 63]. Moreover, additional experimental data suggested Kla of certain proteins are related to insulin resistance in human skeletal muscle [64]. Kla is also widely present in the human lung tissues which are under normal physiological states and may be involved in a variety of biological processes such as RNA splicing, actin filament organization, and neutrophil degranulation [39]. In the human brain, Kla of histone is also widely distributed and also plays an important role in epigenetic regulation during neural development by extensively regulating transcriptome remodeling. In 2021, Dai et al. showed that Kla may be present in various nerve cells in the brain [65]. Hagihara et al. identified 63 candidate lactylated proteins in the brain. The experiments also showed that adding lactate and inducing neuronal excitation were both able to stimulate an increase in Kla levels, noting that stress preferentially increases Kla levels of histone H1. However the functional consequences for each lactylated protein remains elusive [66]. By the way, there is evidence to suggest that neurons can uptake up lactate derived from astrocytes which might affect Kla [67]. The lactate could enter plasma membrane through MCTs. This process can be influenced by intracellular lactate concentration and pH gradient, but the lactate concentration is more important. It is the main source for neurons to take up lactate and induce Kla. MCTs belong to the solute carrier 16 (SLC16) gene family and the MCT1, MCT4, and MCT2 are respectively encoded by SLC16A1, SLC16A3, and SLC16A7 [68, 69]. MCT4 might mainly promote the efflux, while MCT1, MCT2 promote influx [69, 70]. Even through, the precise mechanism by which lactate influences the expression of Kla still requires further investigation and research [71].

Kla is also involved in many other physiological processes. For example, in 2022, Yin et al. found that H4K12la and K14 Kla of histone H3 (H3K14la) are associated with microtubule-based movement and cell invasion in *T. gondii* [48]. An et al. demonstrated that Kla is widely distributed in many proteins of F. occidentalis and linked with ribosomes, DNA binding, protein interactions, and cellular metabolism [47]. Wang and his colleagues reported their experimental results which indicated that Kla might change the conformation and structure of gluten. The solubility of gluten was improved because Kla can alter surface charges. The experiments also showed that Kla significantly increased the viscoelasticity of proteins [72]. As investigators dug deeper into PTMs, there is increasing evidences that PTM also plays an important role in prokaryotes. Dong et al. demonstrated that Kla is distributed in prokaryotic *E. coli* and that lactylated proteins are mainly associated with metabolism. They showed that YiaC and CobB are lysine lactylase and lysine delactylase, respectively. They also found that CobB is also able to specifically modulate PykF activity via erasing Kla at K382, which then promotes glycolysis and bacterial growth [29]. In 2023, Shi et al. identified the accumulated lactate and Kla also occurred in maize roots under drought conditions, extending paradigms of lactate and protein Kla from animal immunity to physiology of stress in plants [44].

### Lactylation in complex diseases

#### Inflammation

As we known, increased lactate levels in the tissue microenvironment is also a common feature in chronic inflammation. In the immune system, Kla is also particularly important, especially in innate immune cells, where protein Kla is commonly observed in the monocyte/macrophage system and closely associated with macrophage polarization [23]. Immune cells derived from monocytes in the blood are distributed throughout the body and influenced by many factors. For example, cold exposure is one of the factors that can influence macrophage differentiation. After cold exposure, glycolysis in macrophages is activated, and the differentiation of macrophages is facilitated by histone Kla [22]. While the Kla of protein occurred, the downstream signaling pathways will be activated to regulate inflammatory processes.

During the differentiation of M0 macrophages into M1 or M2 phenotype, there are different patterns of gene expressions. In this process, histone Kla contributes to the plasticity of macrophage by regulating gene transcription [20, 23]. Wang et al. found that H3K18la in bone marrow and circulating monocytes is enhanced rapidly post-myocardial infarction and promoted reparative gene transcription such as IL-10, promoting the regulation of between pro-inflammatory and anti-inflammatory activities, thus favoring a reparative environment and ameliorating cardiac dysfunction [20]. Research also suggested that Kla was elevated when macrophages prepared to die under inflammatory stress and is a consequence but not a cause of macrophage activation [73]. Additionally, Kla also participated in reprogramming pro-inflammatory Th17 cells phenotypes into regulatory T cells, thus suppressing Th17 cell‐driven inflammation and autoimmunity [74].

### Tumor

Metabolic reprogramming is regarded as a hallmark of cancers. In 1923, Otto Warburg firstly observed that cancer cells tended to constitutively carry out glycolysis, even in the presence of oxygen, through Warburg effect, leading to lactate production and accumulation. As a novel PTM derived from lactate, Kla plays important roles in tumor progression, often by extensively remodeling the TME [75]. Anaplastic thyroid cancer (ATC) is an infrequent but fatal cancer. In 2023, Wang et al. explored the relationship between ATC and Kla, and found aerobic glycolysis, producing lactate in ATC cells can increase global protein Kla, among which H4K12la in turn activated multiple gene transcription essential for ATC proliferation. Besides, BRAF V600E could boost glycolytic flux, resulting in H4K12la-driven gene transcription and cell cycle dysregulation. Therefore, the blockade of cellular Kla machinery might synergistically reduce the progression of ATC with BRAF V600E inhibitor [76]. Ocular melanoma is the most common primary ocular malignancy in adults [77]. In 2021, Yu et al. found that accumulated lactate can lead to higher levels of global Kla in ocular melanoma than in normal melanocyte tissues. They also demonstrated for the first time that the crosstalk of histone Kla and m6A methylation in ocular melanoma. Additionally, they showed that H3K18la could positively regulate the transcription of YTHDF2. Then YTHDF2 could recognize the m6A modified PER1 and TP53 mRNAs, and contribute to their degradation. Thus, histone Kla can promote ocular melanoma development by regulating the transcription of YTHDF2 [78].

Interestingly, clear cell renal cell carcinoma (ccRCC) which accounts for 70 to 80 percent of all renal cancers, is also found to be regulated by H3K18la. Mutation of the von Hippel-Lindau (VHL) is an import cause of ccRCC, and inactive VHL positively correlates with the presence of histone Kla in patients. H3K18la promotes the transcription of platelet-derived growth factor receptor β, which in turn facilitates the growth of ccRCC [79]. Similarly, Non-small cell lung cancer (NSCLC) is the most common type of lung cancer [80]. In 2021, Jiang et al. found that lactate can also attenuate glycolysis and maintain mitochondrial homeostasis in NSCLC cells. What’s more, they showed that lactate may regulate cellular metabolism via inducing histone Kla in HK-1 and IDH3G promoters [81].

HCC is the most common type of primary liver cancers [82], accounting for about 80% of primary liver cancers, with a poor prognosis [83]. Cancer stem cells is a small fraction of tumor cells with the ability to influence tumor self-renewal, differentiation and tumorigenesis. Liver cancer stem cells (LCSC) play an important role in regulating the stemness, self-renewal, tumorigenicity, metastasis, recurrence, and therapeutic resistance of HCC [84]. In 2022, Pan et al. discovered that demethylzeylasteral, a triterpene anti-tumor compound, can suppress tumorigenesis of LCSCs via inhibiting Kla of H3K9 and H3K56 [85]. However, the precise mechanism of Kla on the tumorigenesis of HCC is not clear. The first proteomic survey of Kla in HCC (with no metastasis during 3 years of follow-up), normal liver tissues, and lung metastasis samples of HCC was conducted by Han et al. in 2023. They also identified several biological processes in which differentially lactylated proteins are involved, such as amino acid metabolism, ribosomal protein synthesis, and fatty acid metabolism. Additionally, the experiment also verified the Kla levels of two tumor-related proteins: USP14 and ABCF1 [86]. Meanwhile, Yang et al. revealed that Kla preferentially modified enzymes that are involved in metabolic pathways such as TCA and carbohydrate, amino acid, fatty acid and nucleotide metabolism. They further verified the function of adenylate kinase 2 is inhibited by Kla at K28, which in turn promotes the development of HCC [36]. Besides, Jin et al. found that lactylated CCNE2 can promote HCC cell growth, whereas NAD + -dependent deacetylase SIRT3 can reverse this process, which further can induce the apoptosis of HCC cell for suppressing HCC [87]. Moreover, Xu et al. found the royal jelly acid (RJA) exhibited good antitumor effects in HCC cells and elucidated that H3K9la and H3K14la is essential for RJA to exert its antitumor effect [88]. Glypican-3 (GPC3), an emerging drug target in HCC, is highly expressed in the majority of HCC and is associated with poor HCC prognosis [89]. In 2023, Yao et al. showed that GPC3 knockdown can inhibit stemness of HCC cells via attenuating the global Kla levels and c-myc Kla under hypoxia. Therefore, GPC3-mediated Kla might be important to explore GPC3-specific therapeutics in HCC in the future [90].

GC, a leading cause of cancer-related death worldwide, poses a major threat to human health. To explore the functions of Kla in GC AGS cells, Yang et al. conducted a comprehensive analysis of lactylome in GC AGS cells. Notably, they found Kla was more abundant in gastric tumors tissues than adjacent tissues and suggested that high levels of Kla are associated with poor prognosis in GC patients [34]. In addition, pancreatic ductal adenocarcinoma (PDAC), the most intractable and lethal tumors with a 5-year overall survival rate of about 9% [13], is also characterized with severe hypoxia and lactate accumulation. Thereby, it is particularly important to study the relationship between Kla and PDAC. In 2023, Chen et al. found that Nucleolar and spindle associated protein 1 can be lactylated in PDAC cells, which might increase its stabilization to subsequently enhance LDHA-mediated glycolysis and further metastasis of PDAC [14]. Moreover, β-catenin was found to be lactylated under hypoxia in CRC cells, which might further enhance its protein stability to promote the proliferation and stemness of CRC cells [32].

**Fig. 3:**
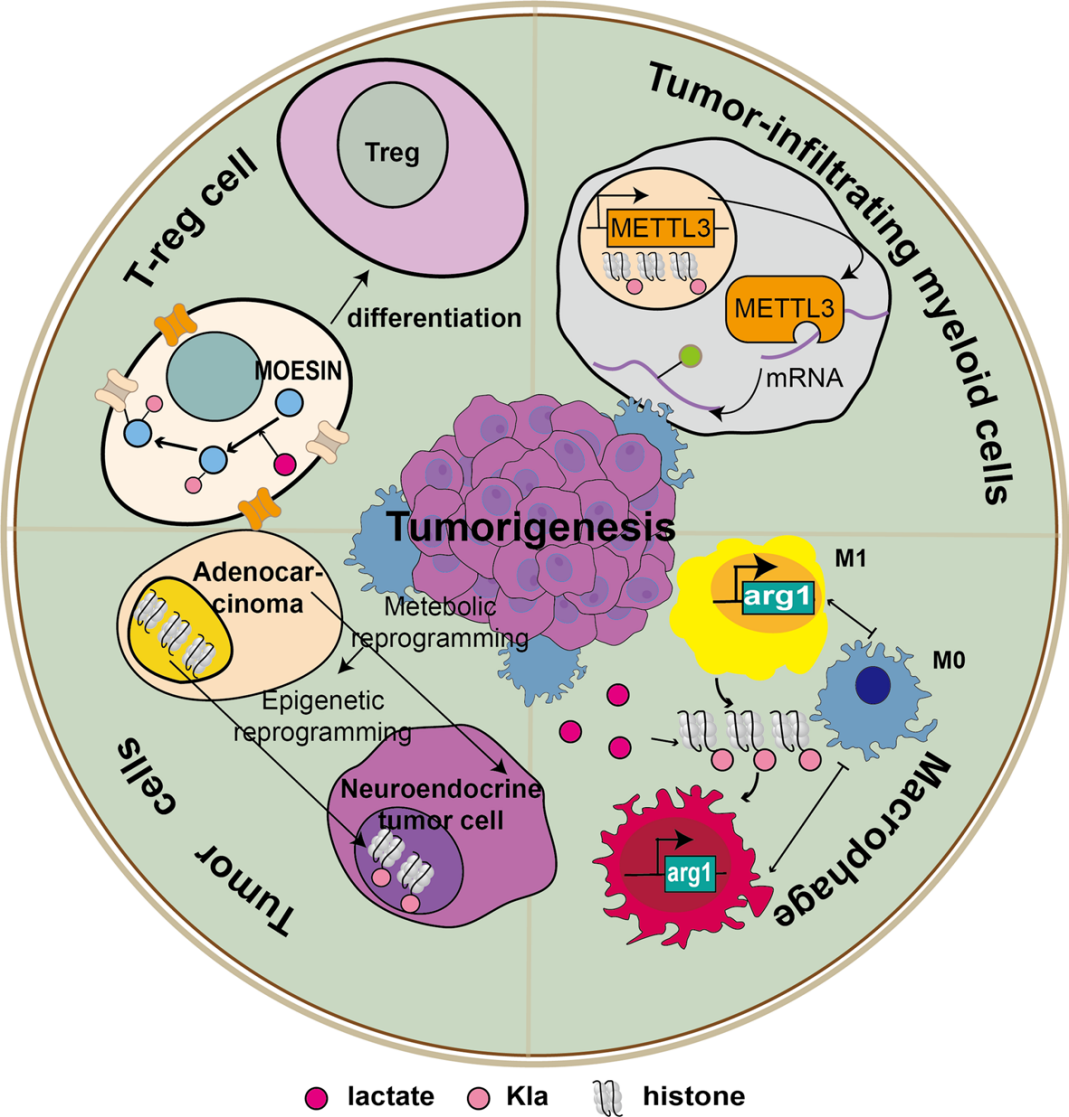
Lactate-derived Kla plays important roles in orchestrating the crosstalk with cancer cells and shaping the TME to participate in tumorigenesis. We presented Kla affects four typical types of cells to participate in tumorigenesis

Moreover, Kla is also found to be important for different immune cells in the TME. For instance, Xiong et al. demonstrated that lactate accumulated in TME potently up-regulate the expression of METTL3 in tumor-filtrating myeloid cells via H3K18la and increased expression of METTL3 in this cell population was correlated with the poor prognosis among patients of colon cancer, whereas myeloid deficiency of METTL3 can attenuate tumor progression and the immunosuppression of TME. Additionally, they identified that lactate can also mediate the Kla of METTL3 at K281 and K345, which was essential for acquiring a stronger RNA-binding capacity and strengthening immunosuppressive functions [91]. Gu et al. demonstrated that lactate can facilitate the Kla of MOESIN at K72 residue to regulate the development and immunosuppressive function of Treg cells, and HCC patients who responded to anti-PD-1 treatment have lower MOESIN Kla levels in Treg cells than nonresponding patients [33]. Using murine cancer models, Zhao et al. found a positive correlation between histone Kla levels and Arg1 expression in tumor-associated macrophages, which further confirmed the positive role of Kla in driving expression of M2-like genes during M1 macrophage polarization [23]. Notably, Kla was found to play crucial roles in regulating cell plasticity and neuroendocrine differentiation in prostate or lung adenocarcinomas. Mechanistically, deficiency in the Numb/Parkin pathway can induce a significant increase in lactate production, which subsequently results in the upregulation of histone Kla and transcriptional expression of neuroendocrine-associated genes [92]. Collectively, lactate-derived Kla plays important roles in orchestrating the crosstalk with cancer cells and shaping the TME to participate in tumorigenesis (Fig. 3).

### Cardiovascular disease

Myocardial infarction and cerebral infarction (CI) commonly occurred in aging population and both of them have high mortality. Recently some researches attach great importance on the influence of histone Kla in myocardial infarction and CI. It is obvious that vascular obstruction might result in local ischemia and hypoxia, which could lead to the accumulation of lactate and induce Kla [19]. Then Kla might participate in the development of these diseases. For instance, lactate exacerbates cardiac dysfunction by promoting endothelial-to-mesenchymal transition (EndoMT), after myocardial infarction via Kla of Snail1. During EndoMT, a process whereby endothelial cells tough various molecular alterations which lead to a phenotypic change toward mesenchymal cells [93]. Mechanistically, lactate increased the Kla of Snail1 and can contribute to its nuclear translocation to bind at the promoter of TGF-β, increasing TGF-β expression, and eventually activate the TGF-β/Smad2 pathway [19]. On the other hand, retinal hypoxia leads to Kla of YY1 in microglial, which then promotes the expression of FGF2, participating in retinal neovascularization and leading to blindness [38]. Additionally, Xu et al. found that Kla of Sox10 is essential for its activation, which leads to macrophage-like vascular smooth muscle cell accumulationand increases vascular inflammation [94]. Cui et al. demonstrated that lactate and Kla plays an important role in the pathogenic phenotype of alveolar macrophages. Mechanistically, lactate can promote histone Kla in the promoters of the profibrotic genes, further enhancing profibrotic gene expression in macrophages and resulting in the pathogenesis of lung fibrosis [95]. Notably, the accumulation of lactate during exercise can enhance Kla of Mecp2, inhibiting epiregulin expression, which in turn might suppress atherosclerosis [17, 18].

### Diseases of the nervous system

Mounting evidences showed that lactate has been produced during glycolysis in brain and its change might be implicated in neuropsychiatric disorders. Alzheimer’s Disease (AD) is a typical type of neurodegenerative diseases, which often cause memory loss and cognitive impairment. Amyloid β (Aβ) plaques and tau neurofibrillary tangles are the two major pathological features in AD [96]. Microglia as an immune cell in central nervous system, is closely associated with Aβ plaques [97]. Pan et al. experimentally found that the levels of histone Kla especially H4K12la are increased in microglia in patients with AD. And this Kla modification is enriched at the promoters of glycolytic genes, which can further activate transcription, and then enhance glycolytic activity and eventually form a positive feedback loop. Therefore, exploring interruption of this positive feedback loop might help the treatment of patients with AD [98]. Additionally, Kla is also found to be enhanced in Schizophrenia model, which can be reversed by a glycolysis inhibitor 2-DG [99]. Moreover, a study suggested that neonatal hypoxic-ischemic encephalopathy might be associated with Kla [100]. Yao et al. identified differentially expressed Kla sites in the CIRI group versus healthy control group, and found many proteins are regulated by Kla. Among these regulated kla proteins, they also confirmed the Kla of Scl25a4 and Slc25a5 in the Ca^2+^ signaling pathway, which might suggest that Kla is also involved in CIRI [40]. Similarly, in ischemic stroke, i.e., CI, the Kla level of LCP1 was clearly enhanced both in vitro and in vivo. Therefore inhibition of glycolysis and reduction of LCP1 Kla levels might mitigate CI [101].

### Other diseases

Furthermore, Kla can be found to be involved in many other diseases. For example, Rho et al. found that HK2-induced lactate can induce H3K18la and further influence the expression of metabolic- related genes, which is required for the activation of hepatic stellate cells and might participate in liver fibrosis. It provided evidence that treating liver fibrosis might be effective by targeting HK2 [102]. In addition, Gao et al. demonstrated that mitochondrial pyruvate carrier 1 can regulate the Kla of fatty acid synthase and the Kla at the K673 site can suppress fatty acid synthase activity, mediating the downregulation of liver lipid accumulation, which might provide a clue to treatment of nonalcoholic fatty liver disease [103].

## Conclusion

As an epigenetic modification, Kla often directly regulates gene expression through various mechanisms. It has been found in both normal physiological and pathological conditions, and is widely distributed in histones and nonhistone proteins. Currently, there have been in-depth studies on Kla in cancer, inflammation, and neurology, but a comprehensive and systematic conclusion is still lacking. Therefore, this review comprehensively reviewed and curated the existing literature to retrieve the newly identified Kla sites on both histones and non-histone proteins and summarized recent major advances toward its regulatory mechanism to help researchers better understand the widespread distribution of Kla and its diverse functions. What’s more, the enzymes directly regulating reversible Kla, often called writers (catalyzing Kla), readers (recognizing Kla), and erasers (removing Kla) were also summarized. Secondly, we also explored and summarized common mechanisms of Kla in normal physiological states. This article then focuses on revealing the effects of Kla on the development of complex diseases, especially in inflammation, tumors, cardiovascular diseases, and neurological disorders. For example, the poor prognosis of colon cancer patients can be correlated with the increased expression of METTL3 in tumor-filtrating myeloid cells, which is regulated by Kla [91] and Kla of MOESIN might make more T cells become Treg cells and promote development of tumor [33]. A good understanding of these processes can help provide important clues for disease diagnosis and treatment, such as in AD the interruption of the feedback loop can help the treatment of patients [98], whereas the Kla of the K673 site may be able to help cure nonalcoholic fatty liver disease [103]. Although the relationship between Kla and other PTMs has not been explored in depth, and has only been hypothesized as competitive, synergistic, and crosstalk [104], it can be affirmed that Kla is an essential balancing mechanism in diverse organisms. We hope our summary of Kla can contribute to a better understanding of this phenomenon and aid in the exploration of novel approaches for the intervention of complex diseases such as cancer.

## Data Availability

Not applicable.

## Abbreviations

Kla: Lysine lactylation
K: Lysine
TCA: Tricarboxylic acid
TME: Tumor microenvironment
PTM: Post-translational modification
MS/MS: Tandem mass spectrometric
LDHA: Lactate dehydrogenase A
Vps34: Vacuolar protein sorting 34
*E.** coli*: *Escherichia coli*
HMGB1: High mobility group box-1
YY1: Yin Yang-1
PCa: Prostate cancer
CRC: Colorectal cancer
ALDOA: Aldolase A
HCC: Hepatocellular carcinoma
CIRI: Cerebral ischemia–reperfusion injury
*T. gondii*: *Toxoplasma gondii*
CCNE2: Cyclin E2
MCTs: Monocarboxylate transporters
SLC16: Solute carrier 16
ATC: Anaplastic thyroid cancer
ccRCC: Clear cell renal cell carcinoma
VHL: Von Hippel-Lindau
NSCLC: Non-small cell lung cancer
LCSC: Liver cancer stem cells
GPC3: Glypican-3
GC: Gastric cancer
PDAC: Pancreatic ductal adenocarcinoma
EndoMT: Endothelial-to-mesenchymal transition
AD: Alzheimer’s disease
Aβ: Amyloid β
CI: Cerebral infarction
